# Mobility recorded by wearable devices and gold standards: the Mobilise-D procedure for data standardization

**DOI:** 10.1038/s41597-023-01930-9

**Published:** 2023-01-19

**Authors:** Luca Palmerini, Luca Reggi, Tecla Bonci, Silvia Del Din, M. Encarna Micó-Amigo, Francesca Salis, Stefano Bertuletti, Marco Caruso, Andrea Cereatti, Eran Gazit, Anisoara Paraschiv-Ionescu, Abolfazl Soltani, Felix Kluge, Arne Küderle, Martin Ullrich, Cameron Kirk, Hugo Hiden, Ilaria D’Ascanio, Clint Hansen, Lynn Rochester, Claudia Mazzà, Lorenzo Chiari

**Affiliations:** 1 University of Bologna, Department of Electrical, Electronic and Information Engineering ‘Guglielmo Marconi’, Bologna, Italy; 2 University of Bologna, Health Sciences and Technologies—Interdepartmental Center for Industrial Research (CIRI-SDV), Bologna, Italy; 3 The University of Sheffield, INSIGNEO Institute for in silico Medicine, Sheffield, UK; 4 The University of Sheffield, Department of Mechanical Engineering, Sheffield, UK; 5 Newcastle University, Translational and Clinical Research Institute, Faculty of Medical Sciences, Newcastle, UK; 6 University of Sassari, Department of Biomedical Sciences, Sassari, Italy; 7 Politecnico di Torino, Department of Electronics and Telecommunications, Torino, Italy; 8 Politecnico di Torino, PolitoBIOMed Lab – Biomedical Engineering Lab, Torino, Italy; 9 Tel Aviv Sourasky Medical Center, Center for the Study of Movement, Cognition and Mobility, Neurological Institute, Tel Aviv-Yafo, Israel; 10 Laboratory of Movement Analysis and Measurement, Ecole Polytechnique Federale de Lausanne, Lausanne, Switzerland; 11 Machine Learning and Data Analytics Lab, Department of Artificial Intelligence in Biomedical Engineering, Friedrich-Alexander-University Erlangen-Nürnberg, Erlangen, Germany; 12 Newcastle University, School of Computing, Newcastle, UK; 13 Neurogeriatrics Kiel, Department of Neurology, University Hospital Schleswig-Holstein, Kiel, Germany; 14 The Newcastle upon Tyne NHS Foundation Trust, Newcastle, UK

**Keywords:** Biomarkers, Biomedical engineering, Databases

## Abstract

Wearable devices are used in movement analysis and physical activity research to extract clinically relevant information about an individual’s mobility. Still, heterogeneity in protocols, sensor characteristics, data formats, and gold standards represent a barrier for data sharing, reproducibility, and external validation. In this study, we aim at providing an example of how movement data (from the real-world and the laboratory) recorded from different wearables and gold standard technologies can be organized, integrated, and stored. We leveraged on our experience from a large multi-centric study (Mobilise-D) to provide guidelines that can prove useful to access, understand, and re-use the data that will be made available from the study. These guidelines highlight the encountered challenges and the adopted solutions with the final aim of supporting standardization and integration of data in other studies and, in turn, to increase and facilitate comparison of data recorded in the scientific community. We also provide samples of standardized data, so that both the structure of the data and the procedure can be easily understood and reproduced.

## Introduction

Wearable devices with multi-sensing capabilities (including sensors such as magneto-inertial, contact, pressure, temperature…) are used in movement analysis and physical activity research, both in the laboratory and in the real world. Their primary applications are to evaluate mobility, to extract clinically relevant information, to predict or detect adverse events (e.g., falls), and to evaluate activity profiles, disease’s symptoms (e.g., Parkinson’s disease, multiple sclerosis …), and movement performance^1^. Heterogeneity in data acquisition protocols, sensor models, sensor specifications, location, attachment modalities, data formats, and gold standards (systems providing reference values when validating data from wearable devices) reduce comparability and reproducibility of research studies with such devices and are a barrier for data sharing, re-use, and external validation^2,3^. Appropriate data standardization may overcome such limitations by providing a common framework for different researchers to compare, reproduce, and integrate different studies. It can provide a guide to report important information in a structured way, thus minimizing errors, incomplete information, and missing data. However, very few studies or research projects have investigated these matters or proposed standardization procedures, harmonization protocols, or guidelines based on experts’ consensus. Furthermore, usually wearable devices comprise different type of sensors and are recorded together with other types of data source, such as gold standards or mobile applications. This highlights the importance of data integration, as recently reported by Clay *et al*.^4^, who introduced a new multistakeholder sensor data integration initiative from the Digital Medicine Society. They highlighted the current absence of widely adopted data standards or repositories in this field.

Previous studies have provided suggestions for selection of the monitoring device^5–7^, data collection^2,6–9^, data analysis^2,10–14^, protocol design^2,7,9^, data quality and validity^2,5,7,8,10^, sensor and signal characteristics^5,6,8,9^.

None of those studies focused or provided general solutions for standardization and organization of data from wearable sensors.

The only study, to the best of our knowledge, introducing a procedure to provide a unique standardized format was done by Siirtola *et al*.^3^. They noted that open-access data sets are not always easy to use and to combine as they are often stored in different formats, sensor orientations and measurement units. So, they introduced the OpenHar Matlab toolbox to unify ten publicly available datasets containing data from accelerometers. The toolbox unifies different file formats, measurement ranges and units, activity labels, sampling frequency, and sensor locations. Still, it does not allow to store gold standard data (such as stereophotogrammetric systems) and is not flexible enough to cover different time measurements (e.g., baseline and follow-ups), laboratory protocols, multi-sensor systems (e.g., gyroscope, barometer…).

It is then clear that there is a lack in standardization and a strong heterogeneity in how different researchers store and organize their data in the field of movement analysis using wearable sensors. So, the objective of the current study is to provide a set of procedures and guidelines for data standardization of wearable sensors in movement analysis, both in the laboratory and in real-world. These procedures were implemented and followed to organize, integrate, and store data recorded from multi-sensing wearable devices and associated gold standards, both in the laboratory and in real-world, during Mobilise-D multi-centric studies^15,16^.

Mobilise-D^15^ is a public-private partnership funded by the European Innovative Medicines Initiative 2 Joint Undertaking. The Mobilise-D consortium includes international research partners, pharmaceutical and technical companies. The objectives of Mobilise-D are to develop and validate real-world digital mobility outcomes (DMOs) from wearable sensors. Digital mobility outcomes are mobility parameters used to assess an individual’s health status, such as gait parameters (e.g., walking speed and cadence). These parameters are collected from “digital” system such as wearable devices^17^. Mobilise-D focused on the development of real-world DMOs from a single wearable sensor on the lower back, and on obtaining regulatory approval for such digital mobility outcomes in a variety of disease states. These guidelines can be used to readily access, understand, and re-use the data that will be made available from the consortium, ensuring wide sharing and reproducibility of results. The procedures that will be presented were designed to provide standardized data from the different available datasets that could be efficiently and reliably used for the development and validation of algorithms to extract digital mobility outcomes (DMO), such as real-world walking speed, from the signals recorded by inertial measurement units (IMUs), namely linear accelerations and angular velocities^16^. These procedures were also designed to support the technical^18^ and clinical validation of different DMOs^16^, the subsequent regulatory approval^19^, and clinical adoption^16^. Mobilise-D data will come from a pilot study (Young Adults Reference data, YAR), from the technical validation study (TVS), and from the clinical validation study (CVS)^16,18^. TVS data will be based on 120 participants (healthy older adults, Parkinson’s Disease (PD), chronic obstructive pulmonary disease (COPD), multiple sclerosis (MS), proximal femoral fracture (PFF), and chronic heart failure (CHF) recruited across 5 different sites in 3 European countries^18^. TVS data was recorded from a wearable sensor on the lower back during i) laboratory-based structured activities (e.g., Timed Up and Go, TUG), ii) 2.5 hours of out-of -lab free-living, with a list of suggested activities to implement, and iii) during 7-day real-world monitoring. In i) and ii) there was the availability of gold standards/reference systems for the validation of DMOs (see Mazzà *et al*.^18^ and the Results section for further details). CVS data will be based on a multicenter observational study in 2400 participants from PD, MS, COPD, and PFF cohorts (600 per cohort) recruited in 16 centers across Europe^16^. This data, consisting in 7-day real-world monitoring using a single wearable device on the lower back, will be particularly suited for application of algorithms for the extraction of DMOs (such as real-world waking speed), analysis of patterns, and association with clinical outcomes (such as falls). YAR data is based on 20 healthy young participants, who performed, before the TVS study started, the same activities of the TVS, with the exception of the 7-day monitoring (see the “Example subjects” paragraph of the Results section).

Initial development and validation of algorithms was performed on data from pre-existing datasets (i.e., datasets available from the consortium partners before the start of Mobilise-D, which had similar characteristics to the TVS, CVS, and YAR datasets). These datasets were recorded by one or more IMUs, possibly with the availability of a corresponding gold standard (e.g., a stereophotogrammetric system). Since these datasets were recorded by different research groups with different modalities, the proposed standardization procedure allowed to process all these pre-existing datasets (and the Mobilise-D ones) in the same way. Characteristics of the pre-existing datasets can be found in the “Example subjects” paragraph of the Results section.

Accordingly, the guidelines here presented include both sections that are specific to the data recorded during Mobilise-D (YAR, TVS, and CVS) and sections that can be used to standardize all datasets with similar characteristics, to be or already collected. We will highlight the information about these two different types of section when needed. To illustrate in practice how the proposed standardization looks like and how it works, example subjects from Mobilise-D and pre-existing datasets are provided, together with code to access the standardized data and to reproduce the standardization procedures (see the “Example subjects” paragraph of the Results section, the Data Availability and the Code Availability sections).

The presented procedures have three aims: i) allowing researchers to easily access all the data that will be available from Mobilise-D enhancing data quality and reducing the workload related to data cleaning and data debugging; ii) allowing researchers to directly standardize their own data iii) support future studies of standardization of wearable sensors in movement analysis.

## Results

Pre-existing datasets, the YAR, and the TVS datasets were standardized according to the presented procedure. The CVS dataset will also be standardized using the presented procedure within future work of the Mobilise-D consortium. Below we provide an overview of the five main domains that we considered for standardization, finishing with a description of the standardized examples we are providing to illustrate the approach. The five main domains that were identified for standardization are:

1. File format and Data Structure
2. Sensor locations and orientation convention of sensor signals
3. Measurement units and sampling frequency
4. Timing references
5. Gold standards

In the following, all the details, explanations, and standardization procedures related to these domains can be found.

### File format and data structure

We chose the *.mat* Matlab file format. The data structure is composed of a folder for each subject in the dataset. In Mobilise-D datasets, subject folder names are progressive IDs consisting of four digits (*snnn*), where *s* is the clinical site and *nnn* the number of the participant acquired in each site (e.g., 1001 represents the participant 001 recorded in the clinical site 1). Instead, in pre-existing datasets, which were standardized according to these guidelines, usually the original IDs assigned to the subjects in the relevant project (or other names, to allow flexibility) were used as folder names.

**Fig. 1:**
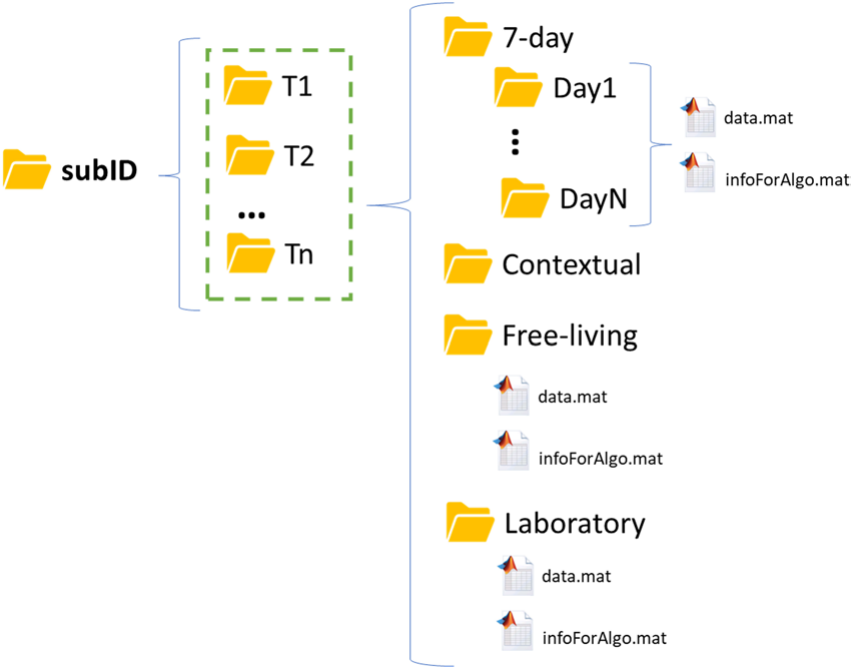
Structure for subject folders. The division in different time measures (T1,T2,…Tn) is only made for the Mobilise-D CVS study.

Within each subject folder, there can be several folders. In the right part of Fig. 1, we have the four main folders used to save the data acquired in Mobilise-D datasets: *7-day*, *Contextual*, *Free-living*, and *Laboratory*. The *7-day*, *Free-living*, and *Laboratory* folders have two *.mat* files inside (the 7-day in each of its subfolders). The first one is the *data.mat* file, which is the core structure of these guidelines, containing wearable device and gold standards data. Each *data.mat* is related to a specific experiment recorded by wearable devices. The *7-day* folder is related to real-life 7-day monitoring, the *Free-living* folder is related to a 2.5 hours out-of-lab recording containing unscripted free-living activities, and the *Laboratory* folder is related to laboratory walking tests. Further details on these protocols can be found in^18^. The second file, *infoForAlgo.mat*, contains the information necessary to run (some of) the algorithms, such as anthropometric measurements or the presence of walking aids (see Supplementary File 1 for further details).

This data structure may also contain other files or folders that are not the focus of these guidelines. As an example, the *Contextual* folder (Fig. 1), contains data related to contextual factors^18^ (e.g., weather and indoor position) recorded during Mobilise-D 7-days monitoring. As a further example, unstandardized raw files recorded by the wearable device may be present (e.g., the *.csv* files of acceleration and angular velocity from the DynaPort MM+ (McRoberts, The Hague, The Netherlands) device used in both TVS and CVS).

Two divisions into subfolders are presented in Fig. 1. The first list of subfolders T1,…, Tn is intended only for the CVS study where there will be several time measurements (T1 = first evaluation, T2 = second evaluation…, Tn = n-th evaluation). The second division (Day1, …, DayN) is used for the 7-day monitoring (TVS and CVS), where the whole acquisition is split into seven different days going from midnight to midnight (local time). These two divisions were made to split the total expected size of a single *data.mat* in more smaller files, thus facilitating and improving algorithm development and testing.

The *data.mat* file can have two different structures, which reflect the two main types of data of interest to Mobilise-D recorded by wearable devices. In general, these two data types encompass a wide range of experiments in functional assessment and monitoring.

1. Data recorded with a detailed protocol, made of different tests. Each test can involve more trials (i.e., repetitions). As an example, the protocol could be made of 4 tests: Standing Posture, Timed Up and Go, Straight Walking at comfortable speed, and Straight Walking at fast speed. Each of these tests may have different trials (repetitions). This is usually done for example to evaluate the reproducibility of the test, or to obtain average values, or to evaluate differences among the performed repetitions.
2. Data recorded in unstructured recordings, without a specific protocol instructing the subjects to perform specific actions. As possible examples there are 7-day real-world monitoring periods (the subject performs her/his daily life activities without instructions or specific constraints), and sessions of unscripted free-living activities, which can be performed in the laboratory or outside.

We made this distinction to differentiate two conceptually different experimental designs and to improve the availability and processing of the data. As an example, it is possible to create a procedure to use when there are tests with different trials (e.g., selecting the average of the performed trials, or discarding the first trial…).

Consequently, the *Laboratory* folder contains a *data.mat* of type 1, while both the *7-day* and *Free-Living* folders contain a *data.mat* of type 2.

**Fig. 2:**
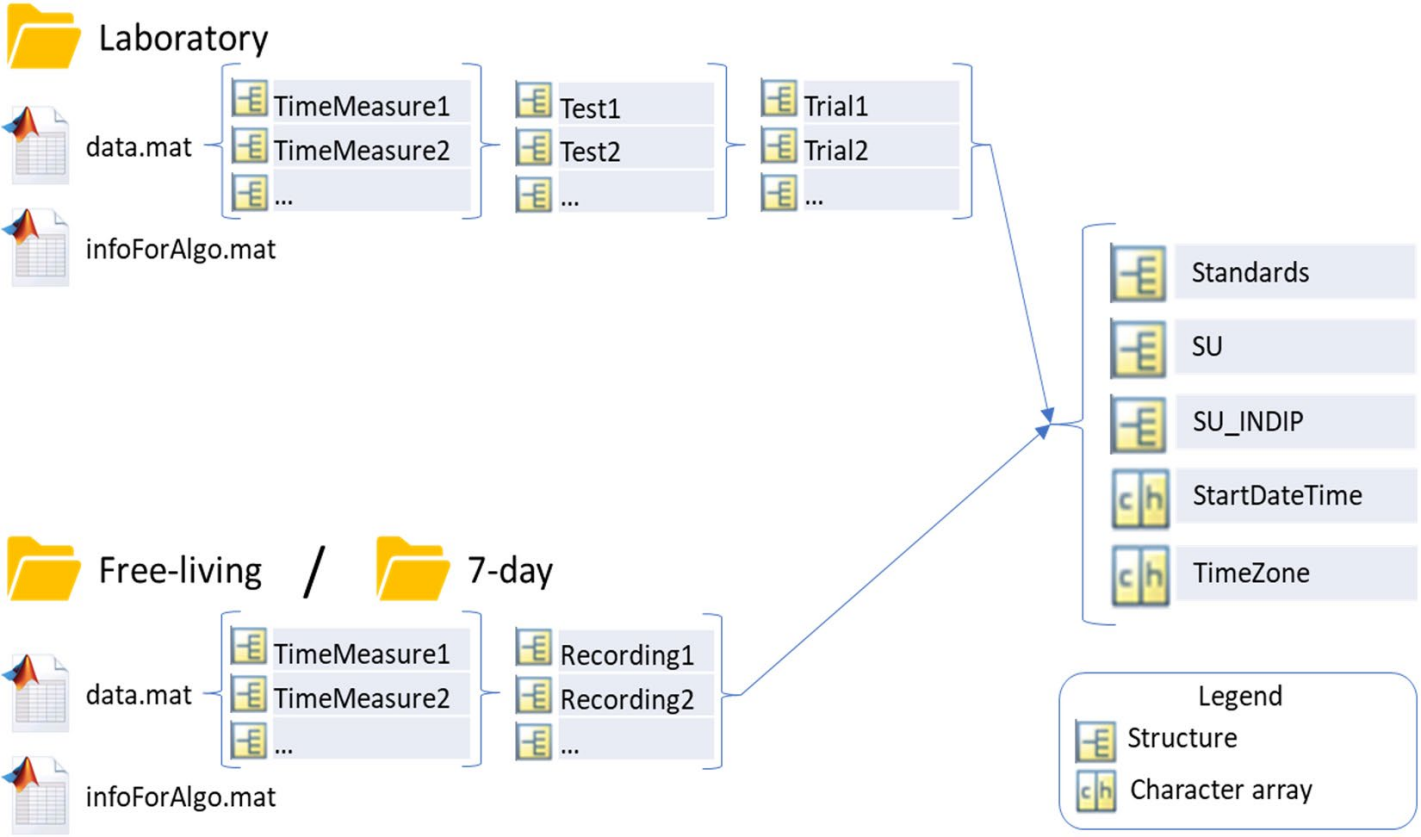
The two types of structure of *data.mat*.

The structure of *data.mat* for the two types of data organization is shown in Fig. 2. Both data types are composed of different levels to manage the possible presence of multiple time measurements. For Type 1, for each time measurement there are two levels: tests and trials. Trials can be different repetitions of the same test (e.g., two repetitions of a 10-meter walking test). Type 2 has a single level, that covers the whole recording in that time measurement (e.g., 24 hours monitored at the baseline time point of a clinical study).

The raw signals from the main wearable device (in Mobilise-D it is the one that is used both for the Laboratory assessment and for the real-world continuous monitoring) are saved within the Sensor Unit (SU) field depending on the location. If more than one device type is used, there will be several SU fields named “SU_DeviceName”. For example, for the Mobilise-D trials, either the DynaPort MM+ or the AX6 (Axivity, York, UK) is placed on the lower back and is referenced as SU in both the TVS and CVS studies. In addition to that, in the TVS study, there is the INDIP (inertial module with distance sensors and pressure insoles) system^20–23^, which is used in both laboratory and free-living acquisitions, where it is used as a reference system. It comprises, among other things, four IMUs. In this case, we saved Mobilise-D main sensor (MM+ or AX6) in SU and the four INDIP sensors in SU_INDIP. In this case, with four sensors of the same type (same brand and model) in four different locations, there will be only one SU_INDIP with the four corresponding locations.

**Fig. 3:**
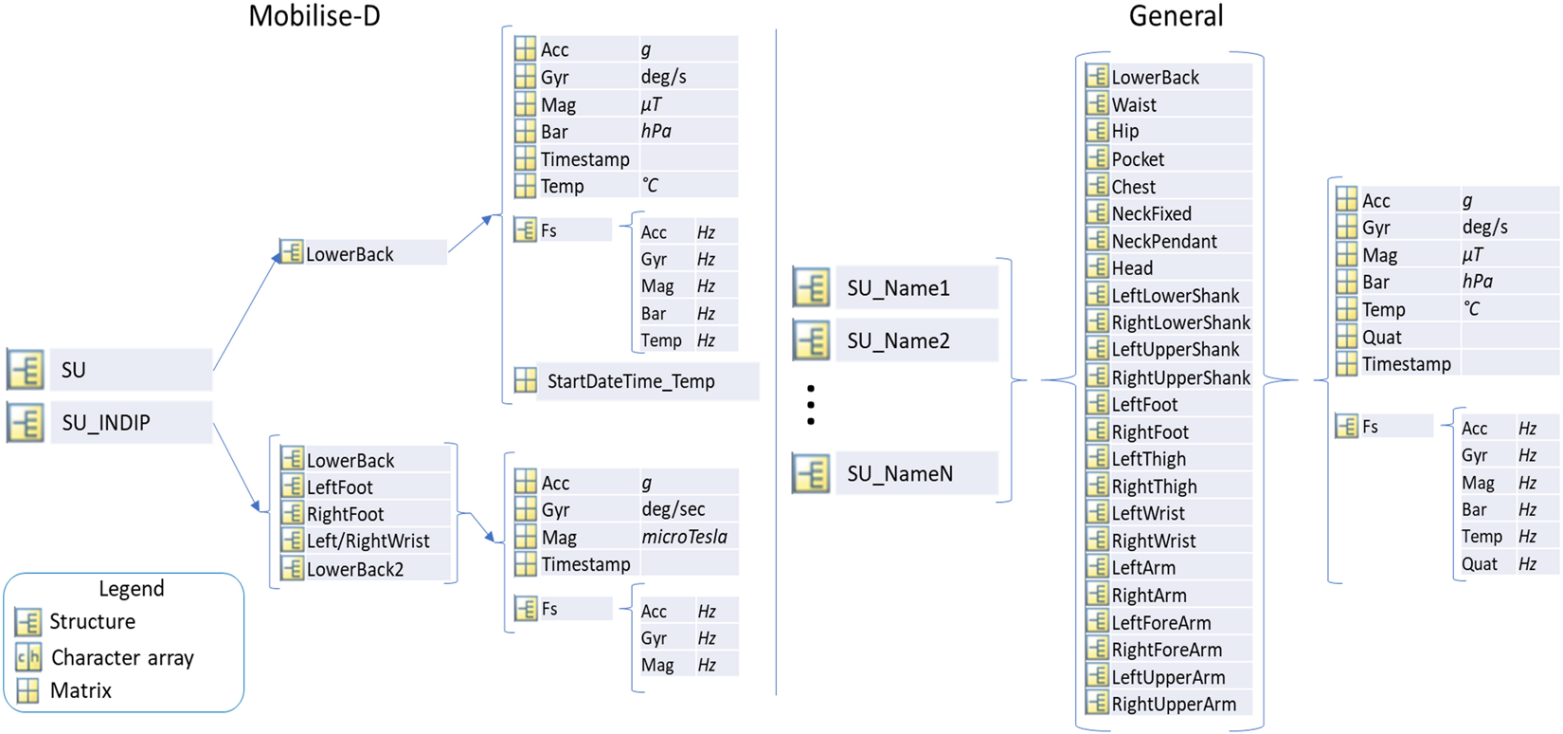
Structure of the data inside the sensor units (SU), for the Mobilise-D studies and for more general datasets (e.g., pre-existing datasets). Signals from accelerometer (Acc), gyroscope (Gyr), magnetometer (Mag), Barometer (Bar), and Temperature Sensor (Temp)  are saved in the Mobilise-D structure (on the left). Additional signals, such as quaternions (Quat), may be saved in the general structure (on the right).

All the signals measured by each device are saved with the structure shown in Fig. 3, following the standardization rules about orientation and unit of measurements (see next paragraphs). The field “Fs” contains the sampling frequency of all the sensors allowing to keep track of different sampling rates.

Within the “Standards” field, there is the processed output of the gold standards present in the dataset and, when available, their associated raw signals (see Fig. 7 and the paragraph about Gold Standards).

### Sensor locations and orientation convention of sensor signals

In Mobilise-D, the selected sensor (SU) location for the 7-day recordings (in CVS and TVS studies) was the lower back, with the Dynaport MM + device or the AX6 device^16,18^. The sensor used and the related different attachment modalities, body-worn and body-attached respectively, are reported in *infoForAlgo.mat* (see Supplementary File 1).

In general, further detailed information about the attachment modality (e.g., use of a specific belt for body-worn and use of tape for body-attached) are present in the protocol information of the related study, which is in the *Dataset Info* file for pre-existing datasets (see Supplementary File 3) and for the Mobilise-D pilot study (YAR) and will be shared in the future for Mobilise-D studies.

In the TVS and YAR studies, the main sensor (SU) location was the same, while the INDIP locations were lower back, left and right foot, and non-dominant wrist. Special cases may occur in the Laboratory sessions of TVS, such as the “LowerBack2” location (see left part of Fig. 3), where the SU_INDIP sensor on the wrist was moved to the lower back to test whether the type of attachment can influence the algorithm robustness in the estimation of DMOs. In this case, two units of the same type (SU_INDIP) were placed in the same location with different attachments (i.e., one by an elastic belt while the other was directly attached to the skin).

In the right part of Fig. 3 (“General”), a wider list of possible locations is presented, which was implemented based on the pre-existing datasets that were available in the consortium (and were standardized) and on the most used locations in the literature for similar experiments. The naming of the locations is always a single string with capital letters for each word forming the string (e.g., “LeftLowerShank”). Naming of other locations, following the same naming conventions, could be added for further future datasets.

**Fig. 4:**
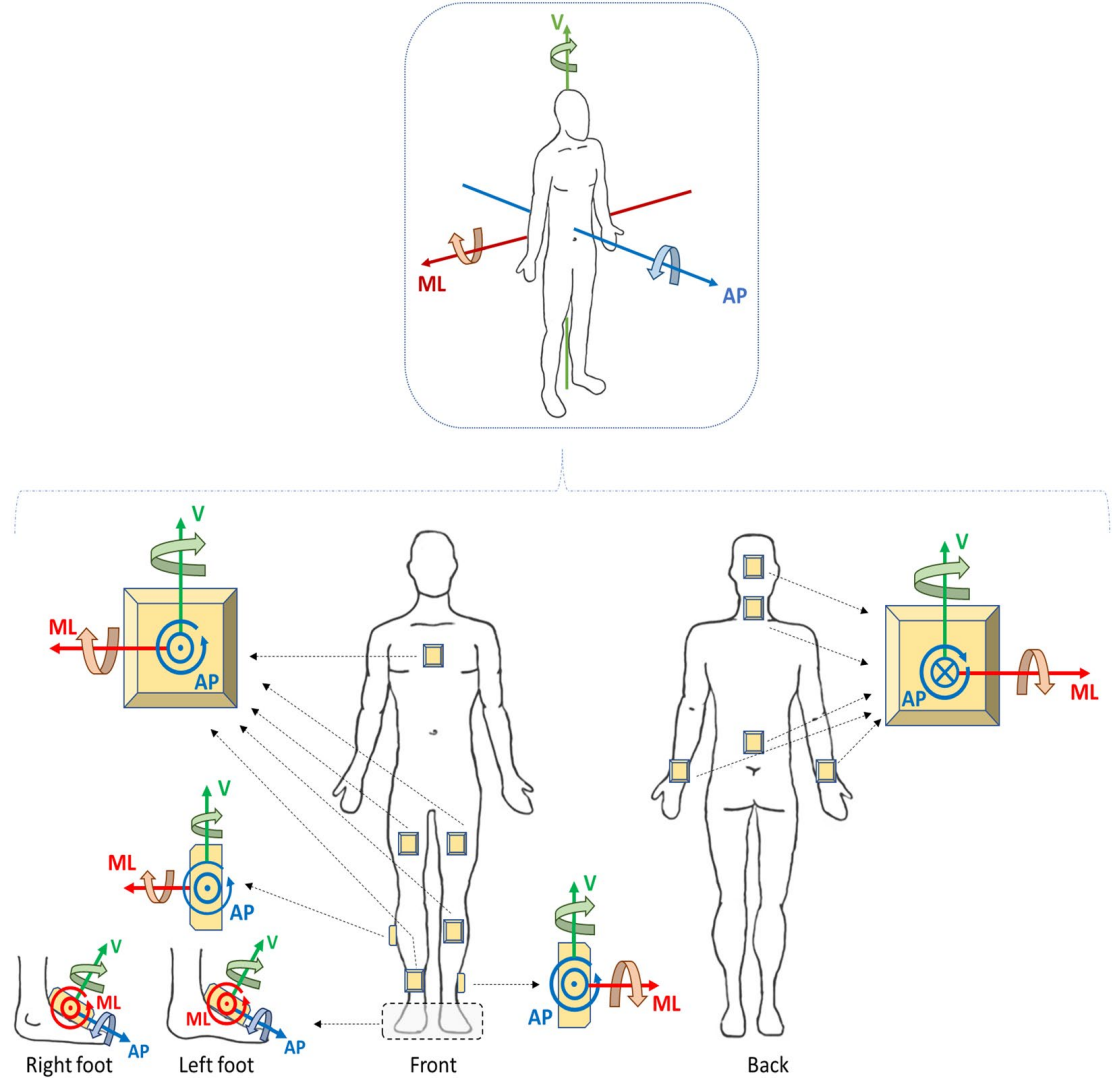
General convention on orientation of recorded signals (top) and examples related to specific locations (bottom). In the detailed part of feet sensor orientation, both feet are seen from an observer on the left of the person.

Another important aspect is the orientation convention for recorded signals. In the top part of Fig. 4 we present the adopted general orientation convention which can be used for any sensor location and is related to a standard anatomical position. A sensor placed in any location, considering the standard anatomical position, will have its data transformed (if necessary) for storage so that its axes correspond to the closest standard directions: vertical (V), medio-lateral (ML), and antero-posterior (AP). We used this convention for all the sensors used in Mobilise-D (the main sensor on the lower back, as shown in Fig. 4, and the INDIP sensors) and for the sensors in the pre-existing datasets. We show in Fig. 4 representative examples of how this orientation convention can be used for different sensor locations. The axes in standardized datasets always follow the general conventions in the top part of Fig. 4 and have axes named V, ML, and AP. Positive and negative orientation of axes and of rotations are also shown in Fig. 4, following the right-hand rule. We saved the SU signals in a three-column matrix (acc, gyro, mag in Fig. 3), where V, ML, and AP signals are the first, second, and third columns, respectively. In pre-existing datasets, the SU signals (e.g., acc, gyro) have been re-arranged to follow our convention depending on the original placement of the SUs.

With this convention, as an example, the raw data from the Dynaport MM + device used in TVS and CVS can be input to the structure without changing any axes orientation (i.e., a matrix of three column, with the x,y,z axes by Dynaport MM+, corresponds to V, ML, AP in this guideline), if the wearable device is attached as recommended by both the manufacturer and the protocol guidelines.

**Fig. 5:**
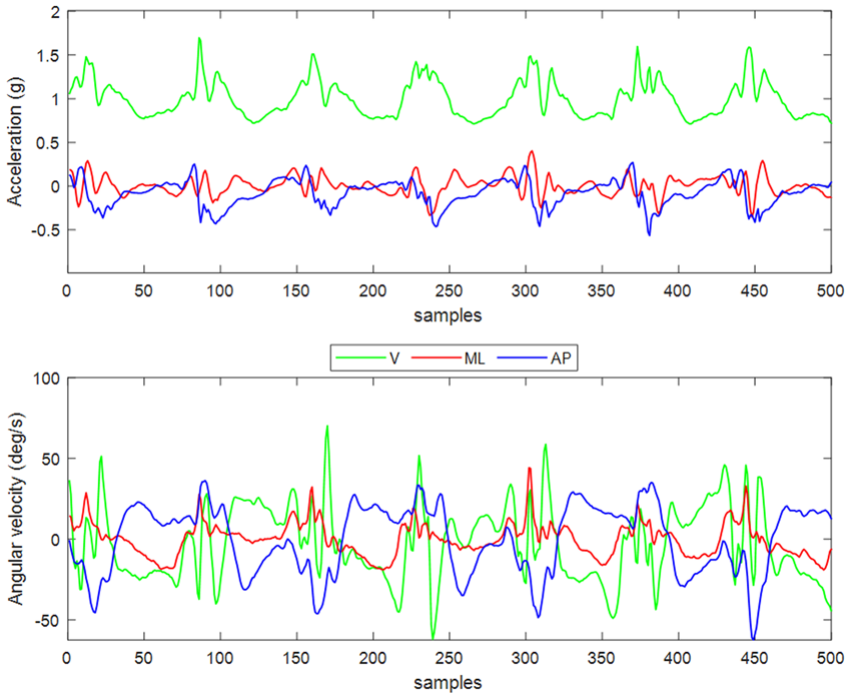
Example of accelerations and angular velocities recorded during walking from the lower back sensor, following the presented convention.

**Fig. 6:**
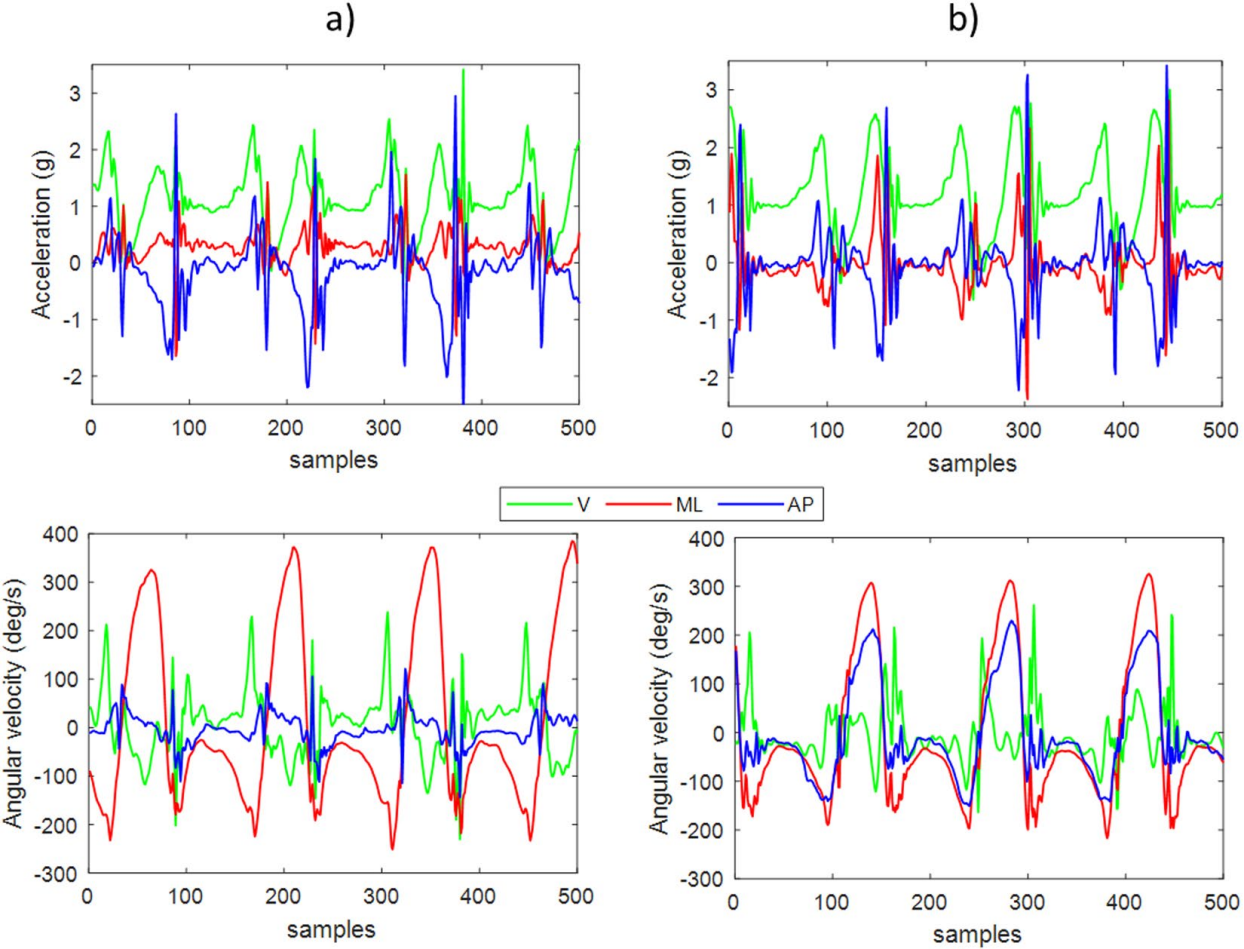
Example of accelerations and angular velocities recorded during walking from two sensors on the left (**a**) and right (**b**) lower shanks, following the presented convention.

Figures 5, 6 present representative examples of what the accelerometer and gyroscope signals would look like when using our convention. Signals are recorded from straight forward walking of a healthy subject of the ICICLE database (one of the pre-exisiting datasets, see the “Example Subjects” paragraph in the result section), using a single sensor on the lower back (Fig. 5) and on each lower shank (Fig. 6).

### Measurement units and sampling frequency

The measurement units for the signals in Mobilise-D datasets are g, deg/s, µT, hPa, and °C for accelerometer, gyroscope, magnetometer, barometer, and temperature sensor, respectively. The guidelines allow storing the sampling frequency of all available sensors.

### Timing references

During Mobilise-D, since different systems are used together in the same acquisition, the problem of how to compare/align the outputs is very important. When a hardware synchronization is not possible or feasible, the use of timing references becomes a valuable resource that allows aligning the data acquired from different devices. During Mobilise-D, this situation occurs because we need to combine the digital mobility outcomes with the confounding factors extracted from the sensor and the smartphone, respectively. As can be noticed from Figs. 2 and 3 there are four fields about the timing references: “StartDateTime”, “TimeZone”, “Timestamp”, and “StartDateTime_Temp”. The field “Timestamp” is a vector with a timestamp for each sample of the signals. Timestamps are saved as Unix, a format that represents the number of seconds that have elapsed since the Unix epoch (00:00:00 UTC on 1 January 1970). They therefore represent the Coordinated Universal Time (UTC). UTC is the primary time standard chosen as a global reference from which all the time zones are calculated, and it is not adjusted for the daylight-saving time. The field “TimeZone” is used to keep the information about the location of the acquisition and allows the conversion from UTC time to local time. This information is saved as a char (character) vector and contains the standard representations of time zone names (e.g., ‘Europe/London’,’Europe/Berlin’,’Asia/Jerusalem’^24^). The “StartDateTime” field is a char vector with the information of the date and local time at which the acquisition started. It follows the ISO 8601 format (‘YYYY-MM-DDThh:mm:ss.SSS ± hh:mm’) and therefore also contains information about the time zone. This field is created from the first timestamp converted in the specific time zone and allows an easy immediate view of the start time, which is especially useful in the 7-day acquisitions where we divide the *data.mat* into seven single days that go from midnight (local time) to midnight (local time). In case the time offset from UTC is zero, then the time zone information (‘±hh:mm’) is replaced with ‘Z’ (‘YYYY-MM-DDThh:mm:ss.SSSZ’). The “StartDateTime” field is also useful in those acquisitions where the timestamp is not provided. In fact, by knowing the start time and the sampling frequency it is possible to recreate the timestamp if needed. For example, this procedure can be used for the temperature in the 7-day acquisition (i.e., if needed, it is possible to recreate the temperature timestamp using the “StartDateTime_Temp” field, which has the same format of “StartDateTime”).

### Gold standards

**Fig. 7:**
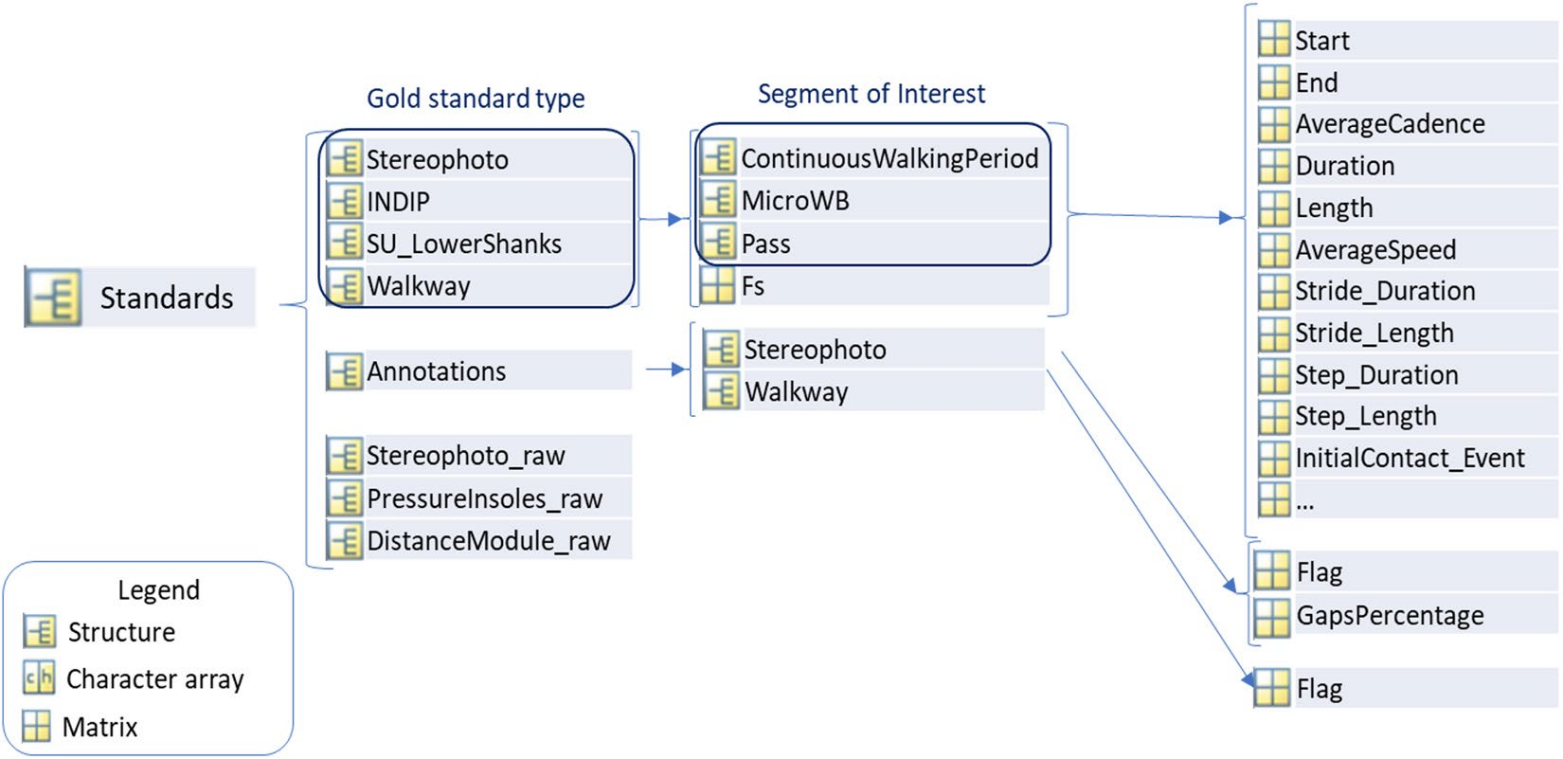
Structure of gold standards.

The “Standards” structure (Fig. 7) contains all the information about the different types of gold standards used as a reference to validate the information obtained from SUs. The fields are identified with the name of the gold standard. In Mobilise-D trials, the gold standards were a stereophotogrammetric system (“Stereophoto”) and the INDIP system^20–23^. In pre-existing datasets, “Walkway” and “SU_LowerShanks”, besides “Stereophoto”, represented the available gold standards. “SU_LowerShank” is based on an algorithm processing data from two IMUs on the left and right lower parts of the shank^25,26^.“Walkway” is an instrumented mat.

The gold standard structure (e.g., “Stereophoto”) contains the related sampling frequency (“Fs”) and the related structures with the processed outputs. Those structures correspond to different segments of interest (walking sequences in this case, since the focus of Mobilise-D is the analysis of real-world walking), based on the specific gold standards. To provide an overview, a “MicroWB” is a walking bout (WB, see definition in^27^) with specific characteristics: it does not contain incline walking, does not contain breaks longer than 3 seconds, and does not contain spurious WBs (sWB = WB with <2 right and 2 left strides). A “ContinuousWalkingPeriod” (CWP) is instead a more inclusive walking period (which can include one or more “MicroWB”s). It can contain “MicroWB”s, incline walking, breaks not longer than three seconds, and sWB. “Pass” is an *ad hoc* structure that is used only for the instrumented walkway because in this case the walking duration only depends on the length of the sensorized part of the instrument. More details about the operational definitions of the presented structures (“MicroWB”, “CWP”, and “Pass”) are beyond the scope of the present work.

“Stereophoto” and “INDIP” both have “ContinuousWalkingPeriod” (CWP) and “MicroWB”. “SU_LowerShanks” only has “CWP” and “Walkway” only has “Pass”.

“CWP”, “MicroWB”, and “Pass” are 1xN structures where N is the number of “CWP”/”MicroWB”/”Pass” instances. For each of them a list of standardized parameters is presented. These parameters provide the following information about the identified walking portion (“CWP”, “MicroWB” or “Pass”): general features (e.g., start, end, cadence, length, …), identified turns (e.g., start, end, duration, angle, …), incline walking characteristics (e.g., start, end, duration, elevation, …), stride characteristics (e.g., duration, length, swing, stance, …), detected gait events (initial and final contacts of feet), and the step/stride characteristics (e.g., duration, length, speed, …). The full list of parameters for the different types of walking sequences (“CWP”, “MicroWB”, or “Pass”) and gold standard systems (for Mobilise-D and pre-existing datasets) is presented in Supplementary File 2.

Moreover, in the “Standards” structure, the “Annotations” field allows storing specific information about the gold standards, to better understand their outputs. The “Flag” field is used for the stereophotogrammetry system to show the samples where there is at least a marker occlusion that may influence the validity of the output (see Supplementary File 2 for further explanation). For “Walkway” instead, the “Flag” field is used to show in which part of the IMU signals the subject is on the instrumented mat and whether two outputs are comparable (see Supplementary File 2 for further explanation).

The “GapsPercentage” field is used for “Stereophoto” to have a direct overview of the percentage of samples with marker occlusion compared with the total length of the acquisition.

Finally, the “Standards” structure also allows storing the raw data of the gold standards (if available) in the “NameOfGoldStandard_raw” field. This can be useful in case of unexpected results in the processed output. Some examples of raw data from gold standards are shown in the Supplementary File 2.

### Example subjects

Data from Mobilise-D participants and pre-existing datasets are provided (see Data Availability) as an example of the proposed procedures (see Table 1). In particular:

**Table 1:** List of datasets.

Dataset	Setting	Sensor Locations	Gold standards	Partner or consortium providing the data	Reference
YAR	Laboratory	Lower back	Stereophoto, INDIP	Mobilise-D	^18^
YAR	Free-living	Lower back	INDIP	Mobilise-D	^18^
ICICLE	Laboratory	Lower back, left and right lower shank, head, neck	Walkway, SU_LowerShanks	UNEW	^28,29^
MSIPC2	7-day	Lower back		USFD	
Gait in Lab and real-life settings	Laboratory, Free-living	Lower back, left and right shank	SU_LowerShanks	USFD	^30,31^
MS Project	Laboratory	Lower back, chest, left and right shank	SU_LowerShanks	USFD	^32,33^
UNISS-UNIGE	Laboratory	Lower back, left and right shank	Walkway	UNISS	^20,34^

UNEW: Newcastle University; USFD: University of Sheffield; UNISS: University of Sassari

#### YAR

- One healthy subject with Laboratory and Free-living

#### Pre-existing datasets

- ICICLE: One healthy subject with Laboratory data
- MSIPC2: One MS subject with 7-day data
- Gait in Lab and real-life settings: one healthy subject with Laboratory and Free-living data
- MS Project: one MS subject with Laboratory data
- UNISS-UNIGE: one stroke subject with Laboratory data

Further specific characteristics and differences of pre-existing datasets with respect to Mobilise-D datasets can be found in the Supplementary File 3. Examples of subjects from standardized datasets are available in a public repository (see Data Availability). Notes to help researchers understand more in detail the related standardization procedures can be found in Supplementary File 3.

Ethical regulations for the studies corresponding to the presented datasets were complied. In particular, the YAR pilot study was conducted according the Declaration of Helsinki and was approved by the University of Sheffield Research Ethics Committee (Application number 029143). The ICICLE study had ethical approval from the Newcastle and North Tyneside Research Ethics Committee, and the research was conducted according to the Declaration of Helsinki. All participants signed an informed consent form prior to testing^28,29^. The MSIPC2 study (*Clinicaltrials.gov* NCT03967106) was approved by Yorkshire & The Humber - Bradford Leeds Research Ethics Committee, and the research was conducted in accordance with the Declaration of Helsinki. All participants signed an informed consent form prior to testing. For the study related to the “Gait in Lab and real-life settings” dataset, ethical approval from the University of Sheffield’s Research Ethics Committee was obtained, and the research was conducted according to the Declaration of Helsinki. All participants provided informed written consent^30,31^. Regarding the “MS Project” dataset, its corresponding study was approved by the NRES Committee Yorkshire & The Humber-Bradford Leeds (reference 15/YH/0300). Written informed consents were provided by all participants prior to any testing. The project was conducted in accordance with the Declaration of Helsinki and with local ethical guidelines^32,33^. Regarding the study related to the UNISS-UNIGE dataset, the Declaration of Helsinki was respected, all subjects provided informed written consent, and local ethic committee approval was obtained^20,34^.

## Discussion

These guidelines were designed to follow specific features of Mobilise-D, while trying to be as generalizable as possible for studies (and related datasets) with similar characteristics. Datasets collected before Mobilise-D were standardized using these guidelines, making them comparable to current and future Mobilise-D datasets. Having comparable standardized datasets allowed to efficiently (and reliably) process available data from different datasets for Mobilise-D algorithm development and validation. Besides the specific work of technical validation, the availability of standardized datasets allows to efficiently perform further research analyses, exploiting the re-use of recorded datasets^35^.

Several decisions were made in order to complete the standardization procedure. The decision on the Matlab file format (and related structure) was guided by the following pragmatic aspects: i) it provides a way to store all the needed information with a clear structure, avoiding multiple text or *.csv* files, ii) it allows automatic processing of the Mobilise-D workflows and easy debugging, and iii) it is one of the most used programming languages within the Mobilise-D consortium (i.e., many algorithms for data processing and many pre-existing datasets were available in that format) and in biomedical research studies using wearable sensors^35–40^. Moreover, from a survey among all partners of the Mobilise-D consortium regarding their available pre-existing datasets recorded by wearable devices, it resulted that 39 out of 69 datasets (56.5%) had their data available in Matlab, followed by hdf5, 20.3%, and csv, 11.6%. We are aware there are other possible formats for data storage (e.g., .csv, .json, .hdf5, .txt). Different file formats are in fact used in the fields of physical activity and movement analysis, depending on the brand of the selected devices and the expertise of different research groups on data analysis software (e.g., Matlab, Python, R) and no consensus or proposal for standardization has been provided before. The chosen solution allowed an easy and fast way to store and check data, providing data ready for automatic analysis and for the related workflow in the Mobilise-D cloud platform (e-Science Central^41^). Matlab was also the format that was proposed in the only other scientific work (to the best of our knowledge) presenting a standardization for storage of data from inertial sensors^3^. Furthermore, the use of Matlab format allowed significant memory saving with respect to the original available format of Dynaport MM+ sensor data (*.csv*). In our experience, the memory saving was over 80%, for a single day’s data file. In a representative day of monitoring the total size was in fact 533885 KB for the .*csv* files (one file for each signal was provided) and 97991 KB for the corresponding *data.mat* file. The limitation of this choice is that Matlab is not an open-access software for data analysis. However, it has an open-access alternative (i.e., Octave) and there are routines in main open-access software languages able to open data in a Matlab format, so that the data analysis can then be performed with the preferred software. In fact, we provided a software repository (see Code Availability section) with open-access R and Python codes, so that the *data.mat* files provided in the current work (and future files standardized with this procedure) can be directly loaded either in R or in Python, together with practical examples of loading and plotting signals from the provided example subjects. Finally, we believe that the various aspects and structure capabilities that were highlighted in this work will be useful also for future datasets collected, regardless the different file formats that will be used.

Regarding the orientation convention of sensor signals that we chose, we are aware that many other studies and sensor manufacturers may use different conventions (and no consensus has ever been made on this). We preferred to use V, ML, AP to identify axes in the standardized data instead of x-y-z because it was a clear and quick way to identify them, helping to effectively understand (and check) what kind of movement the recorded signals represent and to compare (and combine) different studies. So, for studies choosing different conventions, we still would like to suggest one important point to consider based on our experience, to avoid errors and to increase future re-use of data: orientation conventions of sensor signals regarding axes, rotations, and positive-negative, should always be clearly reported.

As a further note regarding the adopted procedure, re-alignment of sensors to correct minor deviations from the orientation convention (e.g., due to misalignment of a sensor on the lower back with respect to the vertical axis) was not performed (and should not be performed before storing the data using these guidelines). The signals stored using these guidelines simply had (if needed) a change of axes (and of positive negative directions) so that they could comply with the chosen orientation convention. Still, re-alignment can be considered as a useful pre-processing step when analyzing this type of data.

Regarding gold standards, besides providing a way to organize their processed data, we propose a strategy to deal with possible problems and issues (e.g., missing gait events due to occlusion of the stereophotogrammetric system, see Supplementary File 2) and a way to check whether the gold standard provides reliable information in correspondence with the events identified by the inertial signals (see Supplementary File 2). As a limitation, we focused on gold standard systems that will be present in Mobilise-D trials, but many others are possible (such as video annotation of activities using body-worn cameras), also depending on the aim of the analysis (i.e., on what outcome must be validated). Still, given the flexible structure provided, we believe it is possible to easily add additional gold standards with different outputs (or with a subset of the presented ones). It should be noted that these guidelines were designed to follow specific characteristics of Mobilise-D (type of study, sensor and gold standard characteristics, consortium expertise). They can be used to readily access, understand, and process all the data that will result from Mobilise-D work (more information on https://www.mobilise-d.eu/data and in future works), starting from the example subjects shared with this article.

In addition to this, these guidelines were designed to be generalizable in order to standardize other datasets with similar characteristics. Therefore, they could be directly used by other researchers to format their own mobility data, by following the procedures and examples (or a subset of them) described in this article. Moreover, even in case researchers wanted to implement different standardization procedures (e.g., because of the characteristics of their study/sensors or because of different preferred file formats), these guidelines can still be a useful tool to support standardization, since they show all the challenges and issues that were encountered in such a process, and the corresponding adopted solutions.

Considering then that more accepted standards are available in other research fields (e.g., in biomechanics standardization procedures have been proposed for reporting kinematic data^42–44^ and for the description of soft tissue artifacts^45^; in neuro imaging there are data structures available to organize and describe imaging data^46^), we believe these guidelines could also be a starting point for discussion towards a consensus on a common approach to standardize data from wearable sensors for mobility analysis.

## Methods

The authors of this scientific article are part of the Mobilise-D work package on algorithm development and technical validation. Guidelines were first implemented based on pre-existing datasets (pre-existing to the start of Mobilise-D, available from partners of the Mobilise-D consortium). Pre-existing datasets compatible with the aims of Mobilise-D were identified among consortium partners by using a survey. In this survey, consortium partners were asked to list available datasets potentially in line with the aims of Mobilise-D work. They were asked for datasets recorded by wearable sensors (e.g., IMU), possibly with the availability of a corresponding gold standard (e.g., a stereophotogrammetric system). They were also asked for characteristics of the recording setting (laboratory or real-world), population characteristics, sensor and gold standard characteristics, data sharing policies, and file formats. We did this in order to select the most suited datasets to perform initial algorithm development and validation. The guidelines were then designed, implemented, and used to standardize the selected pre-existing datasets. By doing so, it was possible to perform automatically all the processing Mobilise-D workflow^5,16,18^ on all available datasets, without considering specific dataset characteristics (such as orientation and unit of measurement). Briefly, the Mobilise-D processing workflow requires either laboratory or real-world data from an IMU worn on the lower back, with at least tri-axial accelerometer and gyro signals, and from gold standards to evaluate the performance of concurrent algorithms. The developed algorithms for this workflow extract DMOs (e.g., walking speed, walking bout duration, stride duration, stride length…) from the standardized datasets. DMO estimates from different algorithms can then be evaluated and validated by comparison to the corresponding DMO values provided by the available gold standards.

After being applied to existing datasets, the guidelines were then applied to data collected in a Mobilise-D pilot study that was performed before the TVS (YAR study). Guidelines were then refined based on feedback from algorithm developers and considering the specific characteristics of data collected in the Mobilise-D trials (TVS and CVS)^16,18,19^. This choice was made to have a common data format for both pre-existing datasets and Mobilise-D datasets, so that the processing workflow could seamlessly work on every standardized dataset (pre-existing or Mobilise-D) without any further adaption of the analytical pipelines.

## Supplementary information

Supplementary File 1

Supplementary File 2

Supplementary File 3

## Data Availability

The dataset containing the example subjects reported in the current study is available in the Zenodo repository “Example subjects for Mobilise-D data standardization” (version 1.0.0 upon article submission)^47^. The repository can be accessed at: 10.5281/zenodo.7185429.

## Code availability

The presented data structure and the related example subjects were created in MATLAB R2021b. Example of the data structures in Matlab are available (see Data Availability Section). To illustrate how to standardize raw data, we provide the Matlab code to standardize the raw data of the example subject from the ICICLE dataset (see Data Availability). The code is in the “MATLAB code for standardization” folder in the following GitHub repository (release version v1.0.0 upon article submission): https://github.com/luca-palmerini/Procedure-wearable-data-standardization-Mobilise-D.

The code was used to obtain the standardized *data.mat* and *infoforalgo.mat* of the corresponding example subject. In the same repository, in the “Python code for access” and in the “R code for access”, we provide code to access the standardized data with Python and R programming languages, respectively.
