# Supplementary material for: Mobility recorded by wearable devices and gold standards: the Mobilise-D procedure for data standardization
Source: Sci Data. 2023 Jan 19;10:38. doi: 10.1038/s41597-023-01930-9 (PMC9852581; doi:10.1038/s41597-023-01930-9)
Supplement: Supplementary file 1 [file 41597_2023_1930_MOESM1_ESM.pdf]

Supplementary File 1: Information for algorithms (infoForAlgo.mat)

The file *infoForAlgo.mat* contains information that is required for (some of) the algorithms (extracting digital mobility outcomes, DMO) to run. Such information can be related to demographics, sensor and body characteristics (e.g., height of the sensor), walking aids, and so on.

Supplementary Figure 1 shows *InfoForAlgo* structure. *InfoForAlgo* will only have those fields that have a value for at least one time measurement (i.e., if one of the fields listed above has no value for any time measurements, this field will then not be present). If no information is available for any time measures, then the *infoForAlgo.mat* file will not be present.

Field name	Type	Measurement Unit	Description
Age	Numeric	years	
Height	Numeric	cm	
Weight	Numeric	kg	
SensorHeight	Char	cm	Height from ground to the SU sensor (estimated from other information if not present)
SensorType_SU	Char		'*MM+*','*AX6*','*DP7*'
SensorAttachment_SU	Char		'*Body-Worn*','*Body-Attached*'
ThighLength	Numeric	cm	The length of thigh (from knee to the waist)
KneeHeight	Numeric	cm	Height from ground to the knee
Gender	Char		'*M*' or '*F*'
Handedness	Char		'*R*' or '*L*'
FootSize	Numeric	cm	
WalkingAid_01	Char		0 (WA not used), 1 (WA used)
WalkingAid_Side	Char		'*Bilateral*' (WA on both sides), '*Left*' (WA on the left), '*Right*' (WA on the right), '*Monolateral*' (single unknown side)
WalkingAid_Description	Char		Type of WA used, e.g., '*Stick*', '*Walker*', '*2 Sticks*', ecc
Orthosis_01	Numeric		0 (not used), 1 (used)
Orthosis_Side	Char		'*Bilateral*' (on both sides), '*Left*' (on the left), '*Right*' (on the right), '*Monolateral*' (single unknown side)
Orthosis_Description	Char		Type of orthosis used
INDIP_DataUsed	Char		'*All*' (IMU and PI used), '*PI not used*', '-' (INDIP not provided)
Annotations	Struct		Annotations taken by the operator during the acquisition, Annotations.TextX.TrialY='text'

Supplementary Table 1. InfoForAlgo structure

As a convention that we used, all the fields of the presented table that are present for at least one time measure are filled in the other time measures where they are not present (with the exception of WalkingAid and Annotations fields), as shown in Supplementary Figure 1.

If a value is not present for a time measure and is not numeric (e.g., gender), then the available value is used to fill it. We assume these items (sex, handedness) not to change over time. In case they do, one should use the closest available value (if both previous and following are available then, in order to have a common convention, one should use the previous one).

If a value is not present and is numeric (e.g., age, height), then the average between the original previous value and the original following value is used (i.e., average between TimeMeasure(n-1) and TimeMeasure(n+1)). In case only the previous or only the following is missing then only the available one is used. In case both previous and following values are missing, then the more distant values (step by step) must be considered (e.g., TimeMeasure (n-2) and TimeMeasure(n+2), and so on…).

As a final note, the *infoForAlgo.mat* in each of the Day1…DayN subfolders is the same as the one in the Free-living folder (the sensor is worn during the Free-living evaluation and then the person keeps it for the following 7 days). An automatic copy of the file from the Free-living folder to each of the Day folders was done in order to simplify the workflow running the algorithms.

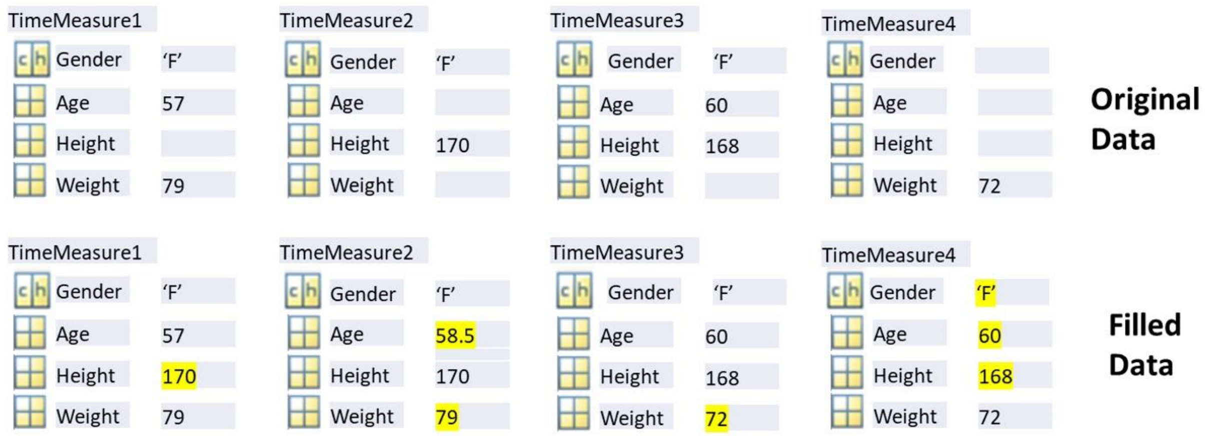

Supplementary Figure 1. Example of filling values for InfoForAlgo
