# Supplementary material for: Mobility recorded by wearable devices and gold standards: the Mobilise-D procedure for data standardization
Source: Sci Data. 2023 Jan 19;10:38. doi: 10.1038/s41597-023-01930-9 (PMC9852581; doi:10.1038/s41597-023-01930-9)
Supplement: Supplementary file 2 [file 41597_2023_1930_MOESM2_ESM.pdf]

Supplementary File 2: Gold standards

Contents

Gold standards 1
List of gold standard outputs 1
Empty and Nan values in gold standard outputs 3
Flag field in Standards 6
Stereophoto 6
Walkway 6
Raw data from gold standards 7

List of gold standard outputs

Supplementary Table 2 is a list of all the gold standard (GS) outputs calculated in the Mobilise-D project that can be found in the “CWP”, “MicroWB”, and “Pass” structures (see the Results section of the main article). Starting from the left, there are the standard names of these parameters, their unit, and a short description. The last four columns on the right show for which gold standard (and related type of walking bout, WB) that parameter will be present. For example, some of the parameters may not be calculated by a specific gold standard (e.g., because the gold standard cannot not calculate that parameter) or may not satisfy the characteristics of the WB type (e.g., incline parameters are not present in MicroWB because the definition of MicroWB does not include incline walking). The parameters that will be present in the corresponding structure (with a tick in the following table) may present empty or NaN values. The former if the event is not present in that WB (e.g., no turns were present in the WB), the latter for calculation exceptions (e.g., a derived parameter such as StrideFrequency is NaN if it was calculated from a Stride_Duration which is made of all NaNs). For meaning and usage of NaN values please refer to the following paragraph. In general, NaN values are used in non-derived parameters (e.g., InitialContact_Event, Step_Duration, and Stride_Duration) to maintain gait sequentiality (e.g., after a left step it is expected a right step) when there are missing values (e.g., using a stereophotogrammetric systems an initial contact (IC) is not found due to markers occlusion in the feet trajectories).

Supplementary Table 2: List of outputs from Gold Standards

Output name	Unit	Description	Walkway	Stereophoto		INDIP		SU_LowerShanks
			**Pass**	**CWP**	**MicroWB**	**CWP**	**MicroWB**	**CWP**
Start	s	Start time instant [I]	✓	✓	✓	✓	✓	✓
End	s	Termination time instant [I]	✓	✓	✓	✓	✓	✓
StrideFrequency	strides/min	$\sum_{k=1}^{n\_stride} \frac{60/Stride\_Duration\_k}{n\_stride}$	✓	✓	✓	✓	✓	✓
Cadence	steps/min	$StrideFrequency * 2$	✓	✓	✓	✓	✓	✓
Duration	s	Duration	✓	✓	✓	✓	✓	✓
Length	m	Traversed distance [II]	✓	✓	✓	✓	✓	✓
LengthFeet	m	Traversed distance [III]	✗	✓	✓	✗	✗	✗
WalkingSpeed	m/s	$\sum_{k=1}^{n\_stride} \frac{Stride\_Speed\_k}{n\_stride}$	✓	✓	✓	✓	✓	✓
AverageStrideLength	m	The average stride length over all detected strides	✓	✓	✓	✓	✓	✓
NumberStrides		Number of identified strides - linked to number of initial contacts	✓	✓	✓	✓	✓	✓
Turning_Flag	FLAG	Indicates if curvilinear portions (1) are present or not (0)	✗	✓	✓	✓	✓	✗
TerminationReason		Identify why a WB has been terminated. This field can be: '**Pause**', '**SharpTurn**', '**InclineWalking**'	✗	✓	✓	✓	✓	✗
Break_Start	s	Start time of each break	✗	✓	✓	✓	✓	✗
Break_End	s	Termination time of each break	✗	✓	✓	✓	✓	✗
Break_Duration	s	Duration of each break	✗	✓	✓	✓	✓	✗
Break_Number		Number of breaks	✗	✓	✓	✓	✓	✗
Turn_Start	s	Start time of each turn [I]	✗	✓	✓	✓	✓	✗
Turn_End	s	Termination time of each turn [I]	✗	✓	✓	✓	✓	✗
Turn_Duration	s	Duration of each turn	✗	✓	✓	✓	✓	✗
Turn_Number		Number of performed turns	✗	✓	✓	✓	✓	✗
Turn_Angle	deg	Angle (magnitude) of each turn	✗	✓	✓	✓	✓	✗
Turn_NumberStrides		Number of strides performed for each turn	✗	✓	✓	✓	✓	✗
Turn_AngularVelocity	deg/s	Angular velocity for each sample of the turn phase	✗	✓	✓	✓	✓	✗
Turn_PeakAngularVelocity	deg/s	Maximum angular velocity measured for each turn	✗	✓	✓	✓	✓	✗
Turn_MeanAngularVelocity	deg/s	Mean angular velocity measured for each turn	✗	✓	✓	✓	✓	✗
Turn_Length	m	Path travelled during the turn	✗	✓	✓	✓	✓	✗
Turning_SharpTurn_Flag	FLAG	Indicates, among the turns, if they are sharp (1) or not (0)	✗	✓	✗	✓	✗	✗
Incline_Start	s	Start time of each incline walking [I]	✗	✓	✗	✓	✗	✗
Incline_End	s	Termination time of each incline walking [I]	✗	✓	✗	✓	✗	✗
Incline_Duration	s	Duration of each incline walking	✗	✓	✗	✓	✗	✗
Incline_Number		Number of detected incline walking	✗	✓	✗	✓	✗	✗
Incline_NumberStrides		Number of strides for each incline walking	✗	✓	✗	✓	✗	✗
Incline_PositiveElevation	m	Positive elevation for each incline walking [IV]	✗	✓	✗	✓	✗	✗
Incline_NegativeElevation	m	Negative elevation for each incline walking [IV]	✗	✓	✗	✓	✗	✗
Stride_InitialContacts		Initial contacts that identify each stride [V]	✗	✓	✓	✓	✓	✓
Stride_Duration	s	Stride or gait cycle duration [VI]	✓	✓	✓	✓	✓	✓
Stride_Length	m	Length, displacement covered within stride cycles	✓	✓	✓	✓	✓	✓
Stride_Height	m	Vertical foot displacement covered within stride cycles	✗	✓	✓	✓	✓	✗

Stride_Speed	m/s	Stride or gait cycle velocity	✓	✓	✓	✓	✓	✓
Stride_TrunkElevationChange	m	Trunk elevations change within strides (vertical displacement)	✗	✓	✓	✗	✗	✗
Stance_Duration	s	Stance phase duration for each stride [VII]	✓	✓	✓	✓	✓	✓
Swing_Duration	s	Swing phase duration for each stride [VII]	✓	✓	✓	✓	✓	✓
Stance_Length	m	Displacement covered within the stance phases	✓	✓	✓	✓	✓	✗
Swing_Length	m	Displacement covered within the swing phases	✓	✓	✓	✓	✓	✗
Stance_Speed	m/s	Velocity of each stance phase [VII]	✓	✓	✓	✓	✓	✗
Swing_Speed	m/s	Velocity of each swing phase [VII]	✓	✓	✓	✓	✓	✗
SingleSupport_Duration	s	Single support (or single limb support phase) duration for each stride [VIII]	✓	✓	✓	✓	✓	✗
DoubleSupport_Duration	s	Double support (or dual limb support phase) duration for each stride [IX]	✓	✓	✓	✓	✓	✓
SingleSupport_Length	m	Length, displacement covered within the single support phases*	✓	✓	✓	✗	✗	✗
DoubleSupport_Length	m	Length, displacement covered within the double support phases	✓	✓	✓	✗	✗	✗
SingleSupport_Speed	m/s	Velocity of each single support phase [VII]	✓	✓	✓	✗	✗	✗
DoubleSupport_Speed	m/s	Velocity of each double support phase [VII]	✓	✓	✓	✗	✗	✗
InitialContact_Event	s	Time instant at which each initial contact is performed	✓	✓	✓	✓	✓	✓
InitialContact_LeftRight		Indicate "Left" or "Right" indicating the side with which the relevant IC is performed [X]	✓	✓	✓	✓	✓	✓
FinalContact_Event	s	Time instant at which each final contact is performed	✓	✓	✓	✓	✓	✓
FinalContact_LeftRight		Indicate "Left" or "Right" indicating the side with which the relevant FC is performed [X]	✓	✓	✓	✓	✓	✓
Step_Duration	s	Step cycle duration [XI]	✓	✓	✓	✓	✓	✓
Step_Length	m	Length, displacement covered within steps	✓	✓	✓	✗	✗	✗
Step_Speed	m/s	Velocity of each step [VII]	✓	✓	✓	✗	✗	✗
Step_TrunkElevationChange	m	Trunk elevations change within steps (vertical displacement)	✗	✓	✓	✗	✗	✗

Notes:

[I]: time instant relative to the start of the recording.
[II]: in “Walkway” it is implemented as X (Last initial contact) - X (First initial contact), where X is the forward direction of movement.
[III]: in “Stereophoto” it is the distance obtained from stride lengths – this info can be used when the “Length” variable is not available (i.e., NaN) to calculate the relevant gait speed.
[IV]: Assessed as the overall positive/negative elevation observed for each elevation walking.
[V]: For each stride it is in the form : [ICstart, ICend]. This field is used to differentiate the meaning of the presence of a NaN in outputssuch as stride duration/length.
[VI]: Time elapsed between consecutive initial contacts of the same foot.
[VII]: Assessed as length/duration of the corresponding parameter.
[VIII]: Time elapsed between the final contact of the current footfall to the initial contact of the next footfall of the same foot.
[IX]: Amount of time spent with both feet on the ground during a gait cycle (for “Walkway” it is Total Double Support: the sum of Initial Double Support and Terminal Double Support).
[X]: A NaN is inserted if the correct side cannot be assessed.
[XI]: Time elapsed from initial contact of one foot to a consecutive one of the contralateral foot.

Empty and Nan values in gold standard outputs

The list of standard outputs varies according to the gold standard (GS) and the type of walking bout (WB), so for each combination of GS and WB, the list is predefined. That said, not always the variables have a corresponding value, but sometimes you may find empty or NaN values. For the empty values the condition is straightforward. A variable can be empty for two reasons: the first is the absence of the measured event in the WB (e.g., no turns found) and the second is because the GS does not calculate that value even if it

could in theory (e.g., by changing the processing algorithm). Regarding NaN, this value is mainly present to keep the synchronization between the different gait features, but it could mean three different things:

Case 1: by convention, all variables related to Single e Double Support, start with a NaN. In the example shown in Supplementary Figure 2, the first value of Single Support should be "IC2-FC0". However, in this way it would contain the Foot Contact before the start of the “MicroWB”/”ContinuousWalkingPeriod”. So, it is assigned a NaN value. The same concept applies for Double Support.

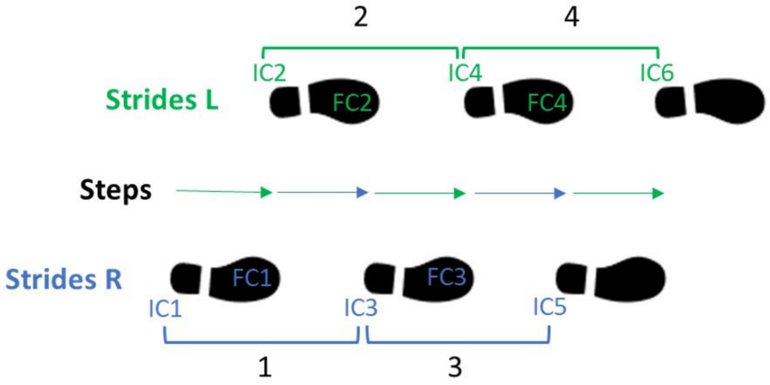

Event	1	2	3	4	5	6
InitialContact_Event	IC1	IC2	IC3	IC4	IC5	IC6
InitiaContact_LeftRight	R	L	R	L	R	L
FinalContact_Event	FC1	FC2	FC3	FC4		
FinalContact_LeftRight	R	L	R	L		
SingleSupport_Duration	NaN	IC3-FC1	IC4-FC2	IC5-FC3		
DoubleSupport_Duration	NaN	(FC1-IC2) +(FC2-IC3)	(FC2-IC3) +(FC3-IC4)	(FC3-IC4) +(FC4-IC5)		

Supplementary Figure 2. Example of Case 1.

Case 2: there is a problem in a GS (e.g., markers occlusion in some trajectories) and the corresponding output is not calculable. Supplementary Figure 3 shows a case where IC8 is unknown due to a problem in the GS (e.g., marker occlusion) and to maintain gait periodicity it was filled with a NaN. For this reason, the strides and steps involving IC8 are NaNs.

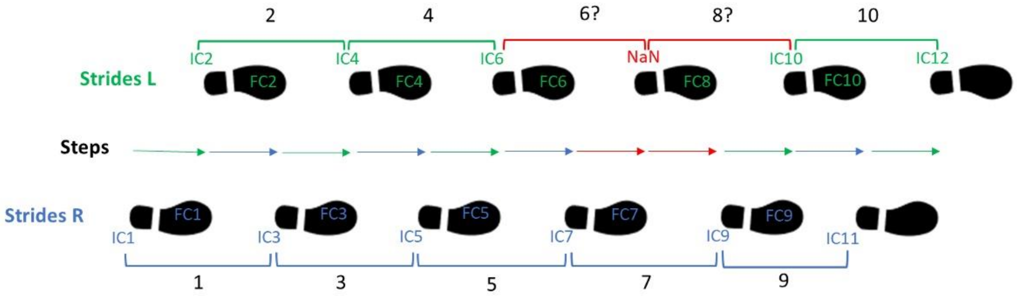

Event	1	2	3	4	5	6	7	8	9	10	11	12
InitialContact_Event	IC1	IC2	IC3	IC4	IC5	IC6	IC7	NaN	IC9	IC10	IC11	IC12
InitiaContact_LeftRight	R	L	R	L	R	L	R	L	R	L	R	L
FinalContact_Event	FC1	FC2	FC3	FC4	FC5	FC6	FC7	FC8	FC9	FC10		
FinalContact_LeftRight	R	L	R	L	R	L	R	L	R	L		
Stride_Duration	IC3-IC1	IC4-IC2	IC5-IC3	IC6-IC4	IC7-IC5	NaN	IC9-IC7	NaN	IC11-IC9	IC12-IC10		
**Stride_InitialContacts**	[IC1,IC3]	[IC2,IC4]	[IC3,IC5]	[IC4,IC6]	[IC5,IC7]	**[IC6,NaN]**	[IC7,IC9]	**[NaN,IC10]**	[IC9,IC11]	[IC10,IC12]		
Step_Duration	IC2-IC1	IC3-IC2	IC4-IC3	IC5-IC4	IC6-IC5	IC7-IC6	NaN	NaN	IC10-IC9	IC11-IC10	IC12-IC11	

Supplementary Figure 3. Example of Case 2

Case 3: we have movements that do not fulfil the stride definition and related thresholds (e.g. minimum duration). In Supplementary Figure 4, strides 5 and 6 do not follow the definition of stride (e.g., stride duration), so the stride 5 and 6 and the steps 6 and 7 are NaN.

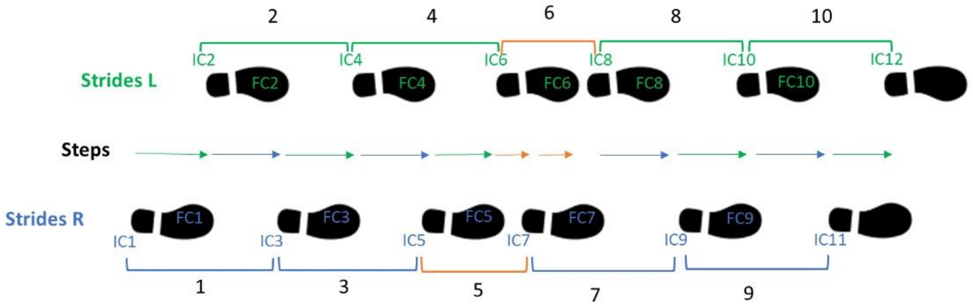

Event	1	2	3	4	5	6	7	8	9	10	11	12
InitialContact_Event	IC1	IC2	IC3	IC4	IC5	IC6	IC7	IC8	IC9	IC10	IC11	IC12
InitiaContact_LeftRight	R	L	R	L	R	L	R	L	R	L	R	L
FinalContact_Event	FC1	FC2	FC3	FC4	FC5	FC6	FC7	FC8	FC9	FC10		
FinalContact_LeftRight	R	L	R	L	R	L	R	L	R	L		
Stride_Duration	IC3-IC1	IC4-IC2	IC5-IC3	IC6-IC4	NaN	NaN	IC9-IC7	IC10-IC8	IC11-IC9	IC12-IC10		
**Stride_InitialContacts**	[IC1,IC3]	[IC2,IC4]	[IC3,IC5]	[IC4,IC6]	**[IC5,IC7]**	**[IC6,IC8]**	[IC7,IC9]	[IC8,IC10]	[IC9,IC11]	[IC10,IC12]		
Step_Duration	IC2-IC1	IC3-IC2	IC4-IC3	IC5-IC4	IC6-IC5	NaN	NaN	IC9-IC8	IC10-IC9	IC11-IC10	IC12-IC11	

Supplementary Figure 4. Example of Case 3

Besides the synchronization, these NaN values are useful to compare GS and IMU outputs, because we know that in those cases we do not have the corresponding info from the standard. To distinguish case 2 from case 3, we added the "Stride_InitialContacts" variable, a vector composed of the two initial contacts (IC) that identify each stride. If one of the two ICs is a NaN, the corresponding NaN in the stride variable is due to case 2, otherwise if both ICs are different from NaN, the corresponding NaN is due to case 3.

Flag field in Standards

The Flag field is used in the “Stereophoto” and “Walkway” standards to check whether the Gold Standard provides reliable information in correspondence with the events identified by the inertial signals. This is important for the validation of algorithms that process sensor data.

Stereophoto

Flag is a 1xN vector, where N is the number of frames of the recorded data, with:

- 0 = if there is at least one occlusion in one of the trajectories used to compute the outputs
- 1 = if no occlusion in any of the trajectories used to calculate the outputs

If a subject is out of the volume of the stereo this value would be 0 since all trajectories would have a missing value. There is no way however to establish with certainty whether the subject is out of the volume, or all trajectories are missing for other reasons (have occlusions). Supplementary Figure 5 shows an example of possible occlusion in the stereophoto trajectories.

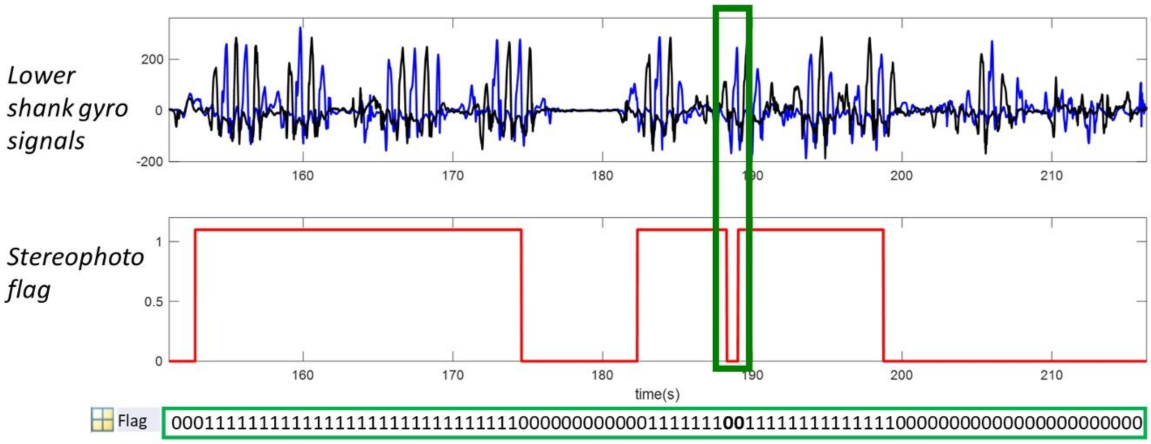

Supplementary Figure 5. Example of an occlusion in the “sterophoto” trajectories

Walkway

Flag is a 1xN vector, where N is the number of samples recorded by the IMUs, with

- 0 = the subject is outside the instrumented mat
- 1 = the subject is on the instrumented mat (this period goes from the first initial contact to the last final contact)

This is important because you can compare IMUs and walkway only when the subject is on the mat (flag = 1), as shown in Supplementary Figure 6.

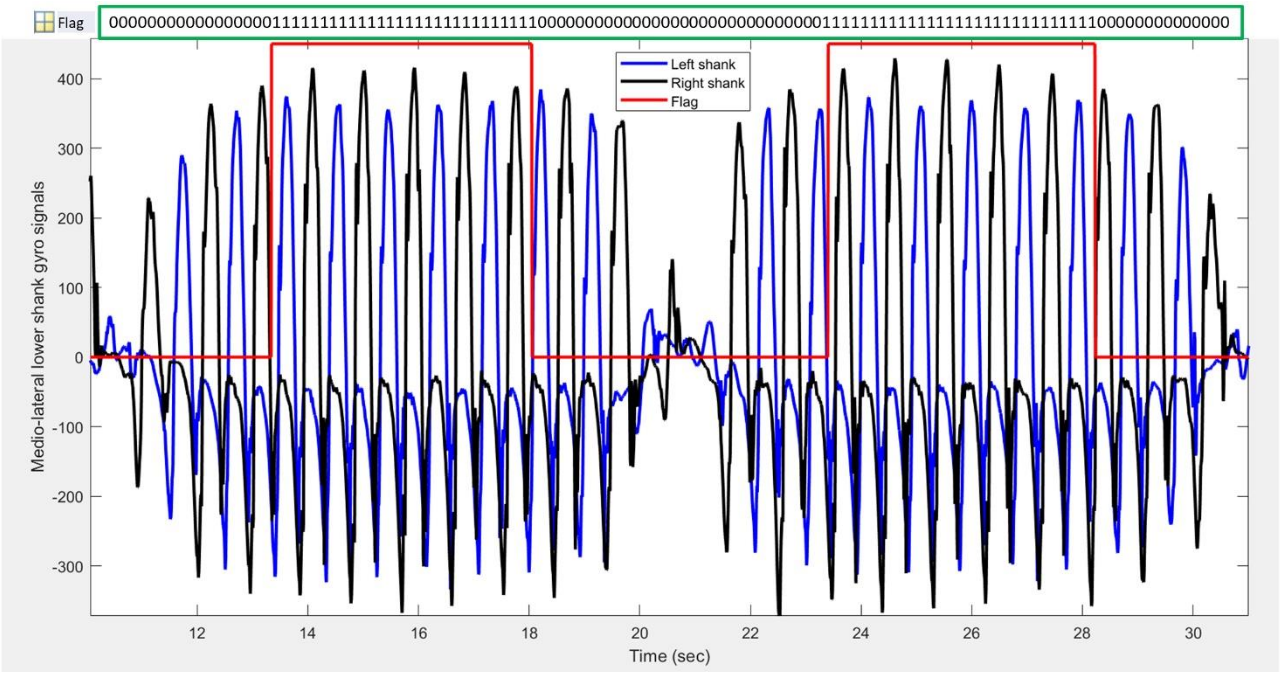

Supplementary Figure 6. Example of the Flag vector for use with the walkway

Raw data from gold standards

The field raw data is not always present, but it can be useful in case of unexpected results in the processed output of a gold standard. In Supplementary Figure 7, three examples of raw data from gold standards saved in the TVS are presented: “Stereophoto”, “PressureInsoles”, and “DistanceModule”. The last two devices are part of the “INDIP” system. Instead, all the raw IMU data, even if they are part of a gold standard (e.g., “INDIP” or “SU_LowerShanks”), are saved in the SU fields (Figure 3 of main manuscript).

For other systems, raw data can also be provided in the original format (file type and/or structure), providing instructions (as a separate file in the dataset info folder) that explain how to use the data. For further studies storing this information, information regarding how the synchronization with the IMU was performed should be provided.

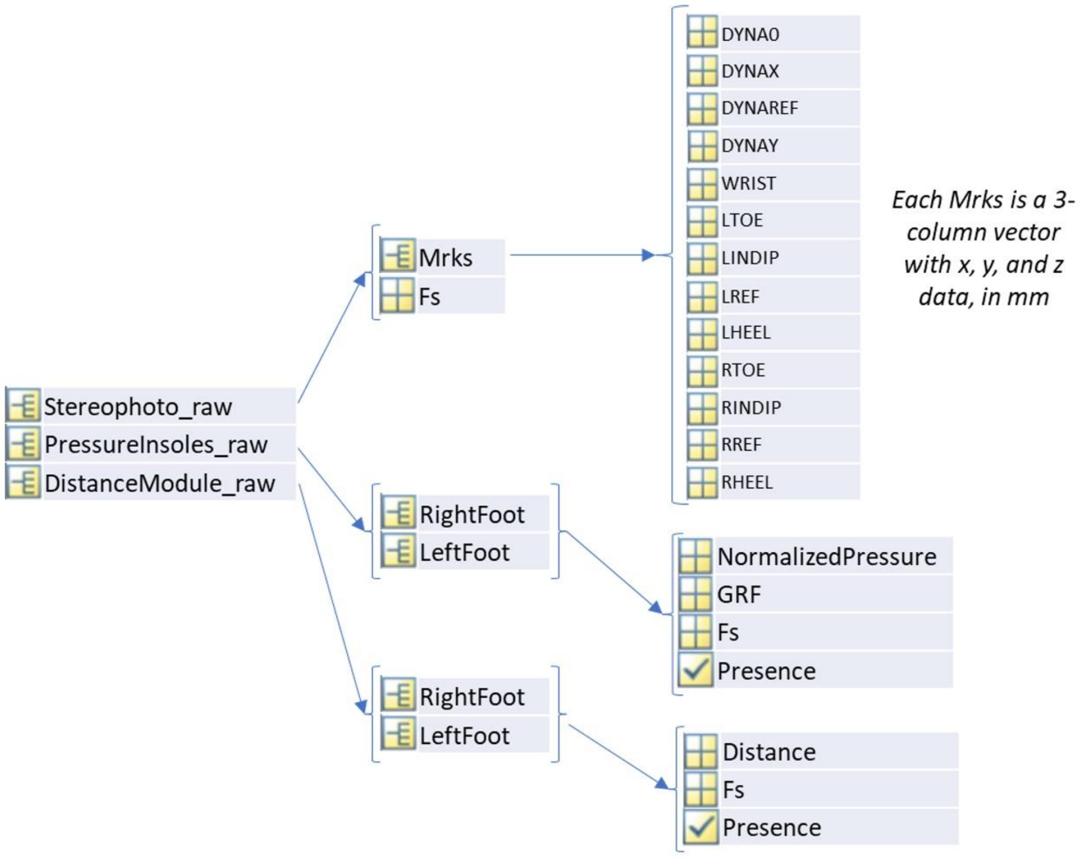

Supplementary Figure 7. Gold standard raw data structure
