# Supplementary material for: Mobility recorded by wearable devices and gold standards: the Mobilise-D procedure for data standardization
Source: Sci Data. 2023 Jan 19;10:38. doi: 10.1038/s41597-023-01930-9 (PMC9852581; doi:10.1038/s41597-023-01930-9)
Supplement: Supplementary file 3 [file 41597_2023_1930_MOESM3_ESM.pdf]

Supplementary File 3: Pre-existing datasets

Contents

Pre-existing datasets........ 1
Dataset Information 1
Clinical Information 1
General practical notes from these guidelines........ 2

For the pre-existing datasets, besides the standardized *data.mat* for each subject, two more documents are provided: *Dataset Info* and *Clinical Info*. These documents contain additional information about the dataset (e.g., participants composition, protocol description, sensors and standards characteristic, and additional notes about the standardization process or exceptions) and about the subjects (e.g., ID, anthropometric information, and clinical scores) respectively. Information about sensor location and orientation is also present.

Dataset Information

The *Dataset Info* is a word or pdf file that describes the dataset and contains the following info (depending on the dataset):

- Participants information: the number of subjects divided by cohorts involved and time measurements.
- Protocol and activities: description of the different tests or trials carried out.
- IMU characteristics: model/brand, sensors specifications (e.g., type of measure, fs, range), positions, attachment modality (e.g., body-fixed with a belt or body-attached with tape) and information about the original orientation.
- Gold Standard characteristic: type, model/brand, fs, and other important info.
- Missing data (if present)
- General notes: about how some parameters are calculated or the presence of particular cases.
- Bibliographic references (if present)

Clinical Information

The *Clinical Info,* which is not the focus of these guidelines, is usually an excel file with the list of subjects that are in the dataset (Supplementary Figure 8). For each subject different information are provided depending on the dataset:

- ID: allows you to identify the folder of the subject considered
- Anthropometric information: e.g., age, gender, weight, height, handedness, …
- Walking aid: presence, type
- Clinical scores (if present): e.g., falls, UPDRSIII, EQ5D, SPPB, …

All the parameters that could change in different time measurements are named as “NameOfParameter_tx”, where x is the time measurement number.

	A	B	C	D	E	F	G	H	I	J	K	L	M	N	O	P	Q
1	ID	Population_t1	Gender	Age_t1	Age_t2	Age_t3	Height_t1	Height_t2	Height_t3	Weight_t1	Weight_t2	Weight_t3	WalkingAid_01_t1	WalkingAid_01_t2	WalkingAid_Side_t2	WalkingAid_Description_t2	WalkingAid_01_t3
2	INGC101	CTRL	M	60.5	62.0	63.6	179	179	179	73.2	73.6	74.2	0	0			0
3	INGC102	CTRL	M	67.3	68.8	70.2	191	191	192	85.2	88.2	85.8	0	0			0
4	INGC103	CTRL	M	87.9	89.3	90.8	181	178	179	96.3	94.6	94.3	0	0			0
5	INGC104	CTRL	M														
6	INGC105	CTRL	F	67.4	68.8	70.4	170	167.5	168	74.5	83	83.4	0	0			0
7	INGC106	CTRL	F	68.1	69.5	71.0	153	154	155	64.2	66	67	0	0			0
8	INGC107	CTRL	F	65.4	66.9	68.4	167	168	169	79.6	77.6	75.4	0	0			0
9	INGC108	CTRL	M	73.6	75.4	76.3	174	172	173	84.4	86.4	84.8	0	0			0
10	INGC109	CTRL	F														
11	INGC110	CTRL	M	60.0			175			66.2							
12	INGC111	CTRL	F	58.6	60.2	61.6	175	173	175	62.8	63	58.2	0	0			0
13	INGC112	CTRL	F	70.6	71.9	73.5	153	155	155	65.6	72	65.4	0	0			0
14	INGC113	CTRL	F	75.0		77.9	170		170	74		77.6	0	1	Monolateral	Stick (only Test2)	0
15	INGC114	CTRL	M														
16	INGC115	CTRL	M	77.3	78.9	80.2	174	175	177	89.8	89	90.8	0	0			0
17	INGC116	CTRL	F	73.6	75.2	76.5	162	162	164	71.8	69.6	69.8	0	0			0
18	INGC117	CTRL	F	68.7	69.9	71.6	160	162	163	74.6	73.8	76	0	0			0
19	INGC118	CTRL	F		76.8	78.0		164	168		66.6	65.4	0	0			0
20	INGC119	CTRL	M	67.2	68.8	70.0	186	184	184	118.7		115.2	0	0			0
21	INGC120	CTRL	F	84.1			159			78							
22	INGC121	CTRL	M	76.7	78.2		184	185		88.4	87.2		0	0			0
23	INGC122	CTRL	F	58.6	60.2		163	163		62.8	64		0	0			0
[illegible]	[illegible]	[illegible]	[illegible]	[illegible]	[illegible]	[illegible]	[illegible]	[illegible]	[illegible]	[illegible]	[illegible]	[illegible]	[illegible]	[illegible]			[illegible]

Supplementary Figure 8. Example of Clinical Info.csv

General practical notes from these guidelines

In this section are listed some further practical notes regarding the standardization procedure, which can be used to understand better the data and to have practical suggestions for recording/standardizing data for similar datasets:

- Orientation convention of sensor signals: In the datasets that we analyzed (collected before Mobilise-D), often the original orientation of sensor signals was different from the one presented in these guidelines. Therefore, a reorientation was needed before saving the signals in *data.mat*. To do this it was necessary for example to swap the axes or change their sign. An important thing to keep in mind is that, if one made some changes in the orientation regarding one sensor (e.g., accelerometer), one should do the same changes to all the other sensors in the same unit (e.g., gyroscope and magnetometer).
- A case that was not considered in these guidelines (because not related to the data that was collected in Mobilise-D) is when the sensor location does not allow a clear understanding of the orientation (e.g., sensor in pocket).
- Empty values: Empty values are for features that are not calculated at all, or that do not present any usable value.
- Nan values: NaN value is present to keep temporal synchronization between different features, as explained in Supplementary Note 2.
